# Reliability of prostate imaging reporting and data system version 2.1 for excluding clinically significant prostate cancer using a 1.5 tesla scanner

**DOI:** 10.1186/s12894-023-01241-6

**Published:** 2023-04-28

**Authors:** Abdallah Sharqawi, Naomi Drye, Abdul Shugaba, Alison O’reilly, Ahmed M. Kadry, A I El-Sakka

**Affiliations:** 1 Urology Dept, Royal Bolton Hospital, Bolton, UK; 2 Urology Dept, Faculty of Medicine, Suez Canal University, Ismailia, Egypt

**Keywords:** Clinically significant prostate cancer, Multiparametric magnetic resonance imaging, PI-RADS score, Trans perineal prostate biopsy

## Abstract

**Introduction:**

Multiparametric magnetic resonance imaging (mpMRI) of the prostate gland is now the recommended initial investigation of choice for the detection of Prostate cancer (PCa). It effectively identifies patients who require prostate biopsies due to the risk of clinically significant PCa. It helps patients with clinically insignificant PCa avoid the invasive biopsies and possible accompanying complications. Large clinical trials have investigated the accuracy of mpMRI in detecting PCa. We performed a local review to examine the reliability of omitting tissue sampling in men with a negative (PIRADS 2 (P2) or less) mpMRI in the primary diagnostic setting.

**Methods:**

This was a retrospective study of patients with clinical suspicion of PCa within a 2-year period. Patients had a mpMRI prior to having trans-perineal prostate gland biopsies. Clinically significant disease was defined as Gleason 7 and above. The descriptive data was analysed using contingency table methods. A p-value less than 0.05 was statistically significant.

**Results:**

Out of 700 patients 90 had an mpMRI score of PIRADS 2. Seventy-seven (85.5%) of these patients had a negative biopsy, 9(10%) showed Gleason 6, 4 patients showed Gleason 7 or above. 78 patients with PIRADS 2 had a PSA density of < 0.15, none of which had a clinically significant biopsy result. The negative predictive value of mpMRI from this study is 95%.

**Conclusion:**

Our results are in line with negative predictive values demonstrated in the current literature. This local study, likely applicable to other district general hospitals, shows that mpMRI is a safe and reliable initial investigation to aid decisions on which patients require biopsies.

## Introduction

Prostate cancer (PCa) is considered the second most common cancer in men and has been found to be underdiagnosed in up to 40–60% of patients [1, 2]. This is due to the inability of digital rectal exams (DRE) and PSA blood tests alone to distinguish between benign and clinically significant prostate cancer. The National Institute for Health and Care excellence (NICE) guidelines recommend Multiparametric magnetic resonance imaging (mpMRI) of the prostate as the first-line investigation for people with suspected clinically localized prostate cancer (PCa) [3]. The evolution of mpMRI has been instrumental in improving prostate cancer diagnosis, as it provides more precise detection and localization of cancerous lesions in the prostate gland. Using mpMRI as the initial investigation allows clinicians to make informed decisions about biopsy and treatment options, resulting in improved outcomes for patients [4]. It has led to increased diagnostic accuracy by selecting patients more likely to inhabit clinically significant PCa, defined as PIRADs 3 and above, for a biopsy, thus reducing the overall number requiring this invasive investigation. Numerous studies have proven its value in the detection of higher-grade cancer [5, 6].

The PROMIS study, which was pivotal in confirming the validity of mpMRI, demonstrated that the use of mpMRI prior to biopsy resulted in 18% more cases of clinically significant PCa being detected, compared with TRUS biopsy alone [6]. Second, in the PRECISION study pre-biopsy MRI resulted in more clinically significant PCa being diagnosed (38 vs. 26%) and a lower detection of insignificant PCa [5]. MpMRI uses T2 sequencing in three planes, plus diffusion and pre-fusion sequences to combine information on anatomy, size, cellularity and vascularity of the tissue to enhance the accuracy of its diagnostic ability [7–10].

MRI prostates are interpreted using the internationally recognized and recommended PIRADS (Prostate Imaging Reporting and Data System) classification which was last updated in 2015 [11]. The higher the PIRADS score represents a higher probability of the presence of PCa. Higher scores are given where there is homogeneous low signal, significant restriction to diffusion and early contrast enhancement [12]. PIRADS has undergone validation in numerous studies and has been broadly embraced by healthcare systems worldwide, such as the National Health Service (NHS) in the UK [3]. The implementation of PIRADS has demonstrated an enhancement in the precision and consistency of the interpretation of prostate MRI, resulting in more dependable and consistent diagnoses of prostate cancer [13]. An investigation by de Rooij et al. uncovered that the utilization of PIRADS led to a noteworthy reduction in the inconsistency among radiologists who were interpreting prostate MRI scans [14].

With the now extensive routine use of mpMRI in PCa the question of its reliability to detect or safely rule out malignancy remains important. Despite the advances, the decision whether to biopsy remains complex. Patients and clinicians must be aware of the risk of both missing significant disease and overdiagnosis. Clinicians should use negative predictive values (NPV) to guide them when they make decisions to not biopsy patients with raised age-specific PSA results. Between 7 and 10% of men will have their clinically significant PCa missed without a biopsy [6].

The aim of this study was to determine the correlation between negative prostate mpMRI (defined as PIRADS 1 or 2) and detection of clinically significant PCa (defined as Gleason 7 or above) within a specific NHS system. This is important in deciding to determine whether it is safe to omit tissue sampling in men with a negative mpMRI in the primary diagnostic setting.

## Materials and methods

This single trust retrospective cohort sectional study was performed from March 2020 to March 2022 at Royal Bolton NHS. Over this period 700 patients had pre-biopsy mpMRIs and trans-perineal prostate gland biopsies when indicated. Included in the study were men referred with a clinical suspicion of PCa who had mpMRI report of PIRADS 2 or less and at least one of the following criteria: persistent elevated PSA and/or suspicious DRE. Only patient fit for all treatment procedures listed in current NICE guidelines were included. The exclusion criteria were prior trans-perineal prostate (TP) biopsy, suspected stage > T2 on DRE within the previous 3 months, serum PSA > 20ng/ml within the previous 3 months, amended upgraded report to P3 or above following multi-disciplinary team (MDT) discussion, contraindication to prostate biopsy, and prior diagnosis or treatment of PCa.

Patients went through mpMRI imaging in a 1.5 Tesla (T) scanner. All scans were run on the same mpMRI protocol. The multiparametric exams included balanced images in T1, T2, diffusion-weighted images (DWI), and dynamic contrast with listed parameters. The scans were reported by an in-house consultant uro-radiologist which were further revised in the MDT discussion following the biopsies irrespective of histology findings.

TP biopsies were performed by trained clinicians in the Urology unit who have been certified to perform the procedure. Biopsies were obtained using a precision point device attached to the rectal ultrasound probe, enabling systematic and targeted biopsies of all cores. Tissue was sent for histological evaluation by unblinded consultant pathologists. Positive samples of prostate adenocarcinoma were classified according to Gleason Score modified by ISUP in 2005. The Gleason grading system was developed to assist in prostate cancer staging and this is scored based on the histopathological architecture of the prostate gland. Scores are provided based on the most common cell morphology and highest grade of cancer seen [15]. Clinically significant disease was defined as the presence of Gleason 4 as primary or secondary pattern, given the associated risk of extra prostatic disease. PIRADS was dichotomized in order to correlate biopsy and MpMRI results.

All collected data were analysed and processed using the statistical package for social sciences version 23 program (SPSS 23). The descriptive data is presented in Tables (1–5). PSA density and family history were analysed using contingency table methods. A p-value less than 0.05 was statistically significant.

## Results

Of the 700 patients who underwent trans-perineal prostatic biopsy and had a pre-biopsy mpMRI, none had a PIRADS 1 score, while 90 had an mpMRI report indicating a PIRADS 2 score. At the time of the biopsy the median age of our participants was 73.5 (range 54–83). Eighty patients had a PSA of less than 10ug/ml, the remaining were between 10-20ug/ml. Regarding PSA density, 78 patients had their PSA density < 0.15, and 12 patients had PSA density ≥ 0.15. The majority of patients (74%) had a white ethnicity and a WHO performance status of 0 (68%). 83% had no family history of prostate cancer, and the mean BMI was 29.2 kg/m2. (Table 1).

**Table 1:** Patient demographics and clinical features

**Age (Median/Range)**	73.5 (54–83)
**Ethnicity**	
White	74
Mixed	3
Asian or Asian British	9
Black or Black British	3
Others	1
**Mean (SD) BMI, Kg/m2**	29.2 (5.4)
**WHO Performance Status**	
0	68
1	22
**Family History of Prostate Cancer**	
Yes	7
No	83
**PSA**	
< 10 ng/ml	80
10–20 ng/ml	10
**PSA Density**	
< 0.15	78
≥ 0.15	12

In terms of biopsy results, 77 patients (85.5%) had a negative biopsy, 9 patients (10%) biopsies showed Gleason 6, 3 patients (3.3%) had Gleason 7 score on histopathological assessment, but only 1 patient had a Gleason score of 8. (Table 2). This equals a 5.5% chance of a clinically significant prostate cancer in the presence of a normal MRI.

**Table 2:** Correlation between PIRADS 2 findings and histopathological Results

Histopathological results	Number	Percentage
Benign	77	85.5%
Gleason 6	9	10%
Gleason 7	3	3.3%
Gleason 8	1	1.1%

Two patients with Gleason 7 disease displayed a 3 + 4 pattern and had clinically significant volume disease, meaning there was more than 50% cancer in any core and more than 3 cores involved, and the cancer was in the transitional zone of the prostate. One patient had a 4 + 3 pattern with less than 50% cancer in any core and less than 3 cores involved, and this was located in the peripheral zone of the prostate. Only one patient had Gleason 8 biopsies with a 4 + 4 pattern, located in the peripheral zone of the prostate. The volume of disease was clinically insignificant; however, the biopsy additionally contained an aggressive variant of prostate cancer in the form of ductal adenocarcinoma. (Table 3)

**Table 3:** Positive biopsies for clinically significant prostate cancer with Negative MRI (PIRADS 2), detailing clinically significant characteristics

Histopathological Characteristics	Gleason 7			Gleason 8
	Patient 1	Patient 2	Patient 3	Patient 1
Tumour Location	Transitional Zone	Peripheral zone	Transitional Zone	Peripheral Zone
Gleason Pattern	3 + 4 (10% pattern 4)	4 + 3 (20% pattern 4)	3 + 4 (15% pattern 4)	4 + 4 (Mixed with ductal adenocarcinoma)
Number of positive prostate biopsy cores	> 3	< 3	> 3	< 3
Percentage of cancer in any positive biopsy core	> 50%	< 50%	> 50%	< 50%

Further correlation of Gleason score to PSA density, for whom had their PSA density < 0.15, most of them had benign biopsy results (72 patients), and 6 patients had Gleason 6 score, but none of them showed Gleason score of 7 or 8. On the other hand, for the patients who had their PSA density of > 0.15, 5 of the patients had benign results, and 3 of them had a Gleason score of 6, while 3 and 1 patients had Gleason score of 7 & 8 respectively (Table 4). Within PIRADS 2 results, the PSA density has a sensitivity of 90.7% and specificity of 100%. Regarding family history of PCa, among the 77 patients who had benign biopsy results, only one of them had a positive family history. Among the 9 patients who had a Gleason score of 6, three of them showed a positive family history. For the two patients with a Gleason score of 7 and positive family history, the positive family history was significant. The only patient with a Gleason score of 8 had a positive family history. (Table 5) The table shows a sensitivity of 95.3% and a specificity of 75%. According to Fisher’s exact test, both tables have p-values below 0.001.

The negative predictive value from this study for mpMRI imaging and the detection of clinically significant PCa is calculated at 95%.

**Table 4:** Gleason score and PSA Density

	PSA Density	
	< 0.15	≥ 0.15
Benign	72(93.6%)	5 (6.4%)
Gleason 6	6 (66.6%)	3 (33.3%)
Gleason 7	0	3 (100%)
Gleason 8	0	1 (100%)

**Table 5:** Gleason score and family history

	Family history of prostate cancer	
	yes	no
Benign	1 (1.2%)	76(98.7%)
Gleason 6	3 (33.3%)	6 (66.6%)
Gleason 7	2 (66.6%)	1(33.3%)
Gleason 8	1 (100%)	0

## Discussion

In our study, 700 patients who had a trans-perineal prostatic biopsy and pre-biopsy mpMRI, 90 of them had an MRI report of PIRADS 2 score. In terms of biopsy results, 77 patients (85.5%) had a negative biopsy, and 9 patients (10%) their biopsy showed Gleason 6 score, and 3 patients (3.3%) had Gleason 7 score on histopathological assessment. Only 1 patient had a Gleason score of 8. We demonstrated a NPV for mpMRI and clinically significant PCa of 95%. This is consistent with a recent Cochrane review which estimated from 12 studies that the NPV of mpMRI for Gleason scores ≥ 3 + 4 = 7 ranges between 86 and 97% [16]. This rate is supported by a meta-analysis demonstrating that men who have negative mpMRI have an approximately 1 in 10 chances of having clinically significant PCa [17].

The two biopsies that showed Gleason 3 + 4 pattern disease had clinically significant volume disease, but the cancer was located in the transitional zone of the prostate, which could explain the false negative findings with mpMRI. A study conducted by Cornud et al. (2017) found that mpMRI was less sensitive for detecting prostate cancer in the transitional zone compared to the peripheral zone. The lower sensitivity in the transitional zone may be due to its complex anatomy and the presence of benign prostatic hyperplasia (BPH) [18]. Another study by Turkbey et al. (2019) evaluated the diagnostic performance of mpMRI for detecting prostate cancer using biopsy as the reference standard. They found that mpMRI had a sensitivity of 60% and specificity of 95% for detecting prostate cancer with a Gleason score ≥ 7. However, the sensitivity was significantly lower for patients with a Gleason score of 3 + 4 compared to those with a Gleason score ≥ 4 + 3, possibly because Gleason score 3 + 4 tumours have a more heterogeneous appearance on MRI and are more difficult to differentiate from benign tissue [19].

The patient with a Gleason score of 4 + 3 pattern of cancer and the patient with a Gleason score of 8 both had low volumes of disease. It’s important to note that low volume disease can be associated with false negative MRI findings. Previous studies have reported that MRI has a low sensitivity of 44% for detecting low-volume prostate cancer and a sensitivity of 82% for detecting high-volume prostate cancer [20, 21]. Additionally, the patient with a Gleason score of 8 was found to have ductal adenocarcinoma (DCa), a rare yet highly aggressive subtype of prostate cancer. DCa can be challenging to detect on multiparametric MRI due to its tendency to show increased signal intensity on T-2 weighted images [22].

In this cohort group, none of the patients with a PSA density less than 0.15 and a negative mpMRI were found to have clinically significant prostate cancer on biopsy. This highlights the effectiveness of using a PSA density cut-off of 0.15 ng/ml/ml or less in the NICE guidelines, as several studies have demonstrated its ability to detect clinically significant prostate cancer while minimizing unnecessary biopsies and associated harms in men with non-cancerous conditions [3, 23, 24].

Trans-perineal biopsies are invasive investigations that carry risks including infection, bleeding and retention [25]. Although the rates of these complications are low and should not deter patients from the investigation when a diagnosis of clinically significant PCa is suspected, they are not insignificant and we need to be sure to council patients correctly on their risk and chance of cancer being detected or missed. This is why establishing the accuracy and reliability of mpMRI imaging is vital. As said previously the NPV of mpMRI is likely to vary between local centres due to multiple factors including cancer prevalence, technique and skill of MRI interpretation and therefore local data is beneficial to guide clinicians and council patients.

There is growing evidence that mpMRI is a safe initial investigation to help select men who do not require a biopsy, avoiding the associated risks, so in 2019 UK NICE and European Association of Urology guidelines suggested the avoidance of biopsies in men with a negative mpMRI, specifically those without high-risk factors [26, 27]. Rozas et al. in 2019 demonstrated that mpMRI was a safe tool to identify patients who should proceed to a biopsy with 88% of negative biopsy also having a negative MRI result and only 2 had clinically significant PCa [28] These again correlate with our results.

In our study 12% of the total biopsies (700) were reported as negative (PIRADs 1 or 2) and could potentially avoid a biopsy. This is less than the results from the PROMISE study [4] but remains a clinically significant number of patients.

The validity of withholding biopsies is strengthened by another large study of 4,259 men, 53% had negative mpMRIs and did not have a biopsy. On follow up imaging 99.6% remained free of clinically significant PCa after 3 years, although only 14% of the original negative patients actually had follow-up scans [29]. So, if patients with negative mpMRI scan are not going to proceed to a biopsy, which men require follow-up and what protocol should we be following? A study followed up 301 men with an initial low suspicion mpMRI with 5-year repeat imaging and demonstrated that overall, 1.7% developed a clinically significant PCa [30]. NICE guidelines recommend repeat PSA at 3–6 months however, in the presence of a high suspicion of PCa, for example PSA density > 0.15ng/ml, High PSA velocity or strong family history, a prostate biopsy should be offered [3]. Those with a low suspicion can be discharged to primary care with PSA at 6 months and advise on when to re-refer.

This study has some limitations mainly as a result of its retrospective design. Firstly, the pathologists analysing the biopsy samples were not blinded to the MRI result and there was no control over the information given on the request forms. Secondly, there was not any active standardisation of who was reporting both the MRI results and the biopsy samples. Although all consultants, the experience and correlation between these doctors was not monitored and assessed. Finally, as we were comparing MRI to trans-perineal biopsy results it is possible some cancers may have been missed as it is known that this type is biopsy will miss a proportion of PCa, although this does represent real clinical experience as not all patients proceed to surgery.

The study findings suggest that using mpMRI as an initial investigation can aid clinicians in determining whether a prostate biopsy is necessary, aligning with NICE guidelines. However, it is important to note that patients with high risk factors, such as a raised PSA density, should still be considered for biopsy. The findings of this study are likely to be relevant for other district general hospitals and can help guide clinicians in their decision-making processes.

## Data Availability

The datasets generated and/or analysed during the current study are not publicly available as it contains all the personal patient’s information but are available from the corresponding author on reasonable request.
